# Between-group competition elicits within-group cooperation in children

**DOI:** 10.1038/srep43277

**Published:** 2017-02-24

**Authors:** Bonaventura Majolo, Laëtitia Maréchal

**Affiliations:** 1 School of Psychology, University of Lincoln, Lincoln LN6 7TS, United Kingdom

## Abstract

Aggressive interactions between groups are frequent in human societies and can bear significant fitness costs and benefits (e.g. death or access to resources). During between-group competitive interactions, more cohesive groups (i.e. groups formed by individuals who cooperate in group defence) should out-perform less cohesive groups, other factors being equal (e.g. group size). The cost/benefit of between-group competition are thought to have driven correlated evolution of traits that favour between-group aggression and within-group cooperation (e.g. parochial altruism). Our aim was to analyse whether the proximate relationship between between-group competition and within-group cooperation is found in 3–10 years old children and the developmental trajectory of such a relationship. We used a large cohort of children (n = 120) and tested whether simulated between-group competition increased within-group cooperation (i.e. how much of a resource children were giving to their group companions) in two experiments. We found greater within-group cooperation when groups of four children were competing with other groups then in the control condition (no between-group competition). Within-group cooperation increased with age. Our study suggests that parochial altruism and in-group/out-group biases emerge early during the course of human development.

Individuals in groups frequently face collective action problems involving, for example, paying taxes, taking part in strike actions or in a conflict against an opposing country or group^1^,^2^,^3^. Collective action problems describe a scenario where each individual has to choose between two alternative strategies, acting selfishly or cooperating with the other group members, which bear both potential costs and benefits for the individual. The benefits are greatest for the group if the more individuals cooperate by contributing to the collective action. In contrast, for any individual, the benefits of cooperation in the collective action only outweigh the cost, for example, of paying taxes, if the other group members do the same and if the benefits of the collective actions are shared equally or according to individual contribution^4^,^5^. Moreover, cooperation increases the greater the likelihood and/or severity of punishment of free-riders^6^. Since the risk of free-riding can rarely be completely ruled out, the most profitable decision (from an economic, cost/benefit point of view) for an individual in a group facing a collective action problem is to not cooperate and act selfishly^2^,^7^,^8^. However, even when acting selfishly would be the most profitable decision, within-group cooperation is still widely observed in many species^9^,^10^.

Aggressive interactions between groups over valuable resources (e.g. food) are observed in many taxa, from ants and birds to human and non-human primates^1^,^11^,^12^,^13^,^14^. The risk of free-riding in between-group competition (BGC) is high as conflicts with other groups can bear significant costs to individual fitness (e.g. injuries or death) but also high benefits (e.g. access to resources or political stability^1^,^3^,^11^). In aggressive BGC, groups composed of a smaller proportion of free-riders should out-perform less cooperative groups (i.e. those with a greater proportion of free-riding individuals), other factors being equal (e.g. group size or fighting ability^5^,^15^). Therefore, under a BGC condition within-group cooperation should increase and the risk of free-riding decrease when the cost of losing a conflict, for each group member, outweighs the benefit of free-riding^16^,^17^,^18^. Following this argument, various disciplines, from evolutionary biology^16^,^19^ and anthropology^20^ to social psychology^21^ and economics^22^, have hypothesised that BGC is positively related to within-group cooperation at the proximate and ultimate level. In non-human animals, mixed support exists for this hypothesis^12^,^13^,^23^. In adult humans, however, cooperation increases under experimental conditions simulating BGC^4^,^18^,^22^,^24^,^25^; but see ref. 26. Moreover, groups composed of individuals who were willing to cooperate with their group companions were more likely to engage in costly between-group interactions^27^. The positive relationship between BGC and within-group cooperation in humans could be due to correlated evolution. It has been proposed that our ancestors faced an extended period of intense warfare, with a high risk of injuries and deaths, during the course of Pleistocene and Holocene^17^,^20^,^28^; but see ref. 29. According to this hypothesis^17^,^20^, the high risk of warfare-associated costs for individual fitness has driven the evolution of a series of cognitive and behavioural traits that favour parochial altruism (i.e. the propensity to be prosocial towards members of own group and aggressive towards out-group) and the recognition of in-group membership, which decrease the occurrence of free-riding when there is an out-group threat. Supporting this view, in-group/out-group biases, fear of out-group, and social norms on fairness and punishment of free-riders have been observed in virtually every human society^30^,^31^,^32^,^33^,^34^.

While there is growing experimental work in adult humans^4^,^18^,^22^,^24^,^25^,^27^, we do not know if a positive relationship between BGC and within-group cooperation is also present in children and, if so, when it emerges during development. In traditional human societies, post-puberty males often are the only sex/age category taking part in fights with other groups/communities^1^. If so, behavioural responses (e.g. parochial altruism or fear of out-group) related to correlated evolution between BGC and within-group cooperation should be stronger or only evident in adult males^35^. However, these behavioural responses to out-group often appear in pre-teenage children of both sexes. Six to eight years old children show in-group/out-group biases, with no gender difference, when asked to predict whether other children would engage in positive or negative social behaviour^36^. Moreover, six year old children are more likely to punish selfish resource allocation by out-group than in-group children^37^. Five to ten years old children are less pro-social towards out-group members when in a BGC condition^38^. Finally, aversion to inequality can be observed in 3–8 years old children and it is strongly related to the development of parochial altruism and in-group/out-group biases^39^. Therefore, ‘sensitivity’ to out-group threat, parochial altruism and punishment of free-riders appear relatively early during the course of human development of pro-sociality, especially from the sixth year of age onward.

The aim of our study was to test two hypotheses. First, we hypothesised that within-group cooperation should increase when groups are facing BGC, in comparison to a control condition (i.e. no BGC), in children, similarly to what observed in adults^18^,^22^,^24^. Second, we hypothesised that the relationship between within-group cooperation and BGC should emerge early in development but should be more pronounced as children get older. This is because children become progressively more prosocial, inequity adverse and more likely to punish free-riders from around the age of six-ten years old^37^,^39^,^40^,^41^,^42^.

We tested our two hypotheses in a large cohort of children (n = 120) between three and ten years old. We asked children to take part in two behavioural economics experiments, both played in groups of four children, using star stickers (see Methods) as the resource at stake. In each experiment, we compared within-group cooperation (i.e. how many stickers children gave to the other children in their group and/or to how many group members) between children allocated to a BGC condition (where BGC was simulated; see Methods) and a control (no BGC) condition. Since younger children (i.e. three to five years old) do not have the same numeracy and mathematical skills of older children^43^,^44^, the aim of experiment #1 was to test our predictions using a simple experimental design (see Methods) that required little numeracy and allowed children to see how resources were allocated among children in their group. In experiment #1, at the beginning of each round we selected one child (i.e. the player) who had to play the game. The player was given three start stickers and had to decide whether they wanted to give any stickers (and, if so, how many) to one or more children in their group and/or how many stickers they wanted to keep for themselves.

Experiment #2, a public goods game, consisted of three rounds. At the beginning of each round we gave each child three stickers: the children had to decide whether they wanted to give any stickers (and, if so, how many) to the common group project and/or how many stickers they wanted to keep for themselves. In experiment #2 our aim was to test if results from experiment #1 could be replicated using a different experimental design requiring more advanced numeracy and rules for the allocation of resources. Moreover, we aimed to compare our findings to data on adults; thus, we used an experimental procedure designed for adults^18^ and adapted it for children.

## Results

Children below the age of six years old have lower numeracy and mathematical skills than older children^44^,^45^ and this may affect the way younger children respond to our experiments. Therefore, in order to rule out the possibility that our results were biased by the different comprehension of the experiments by younger and older children, we first tested our predictions on the whole cohort of children and then repeated the analyses, for our two test variables (experimental condition and age of children), restricted to children who were ≥six years old. This procedure allowed us to see if our results were confirmed when removing younger children.

**Figure 1:**
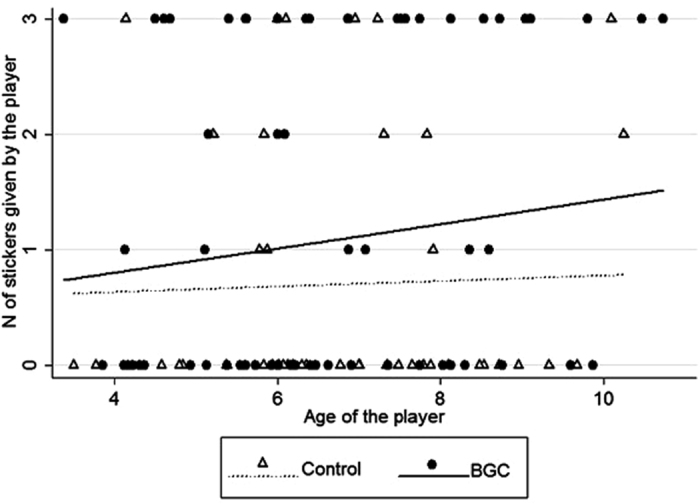
Scatter plot with lines of best fit on the relationship between the age of the player and the number of stickers given by the player, in experiment #1, in the control (triangles and dotted line) or BGC (circles and continuous line) condition.

In experiment #1, the full model for the mixed-model Poisson regression on the number of stickers that the player gave to the other children in their group in each round significantly differed from the null model that only contained the random factor (likelihood ratio test: chi-square (3) = 8.66, p < 0.05). The full model contained our two test variables, experimental condition and age of the children, together with gender of the child as a control variable and Group ID as a random factor (see Methods and Fig. 1). Supporting our first prediction, children were more likely to give stickers to other children in the BGC than in the control condition (Table 1 and Fig. 1; see also Table S2 in the Supplementary Information). Moreover, likelihood to share stickers significantly increased with age of the children (Table 1 and Fig. 1). Gender of the children had no significant effect on the likelihood to share (Table 1). Restricting the analyses to children ≥six years of age confirmed our result for experimental condition (coefficient ± SE = 0.62 ± 0.35, z = 1.86, p = 0.05) but made age of the children non-significant (coefficient ± SE = 0.13 ± 0.09, z = 1.54, p = 0.12).

**Figure 2:**
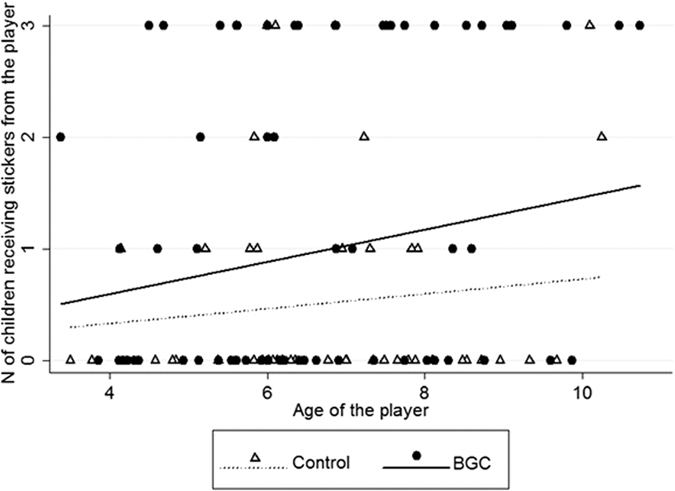
Scatter plot with lines of best fit on the relationship between the age of the player and the number of children receiving stickers from the player, in experiment #1, in the control (triangles and dotted line) or BGC (circles and continuous line) condition.

The full model for the mixed-model Poisson regression on the number of children receiving stickers from the player in each round that significantly differed from the null model (likelihood ratio test: chi-square (7) = 16.19, p < 0.05). The player gave stickers to a larger number of children in their group in the BGC than in the control condition (Table 2 and Fig. 2; see also Table S2 in the Supplementary Information). Moreover, older players gave their stickers to a larger number of group members than younger players (Table 2 and Fig. 2). The control variables had no significant effect on the number of children receiving stickers (Table 2). When we restricted the analyses to children ≥six years of age, the positive and significant effect of BGC on the number of children receiving stickers was confirmed (coefficient ± SE = 0.91 ± 0.25, z = 3.67, p < 0.001). Conversely, age of the children was no longer significant (coefficient ± SE = 0.11 ± 0.08, z = 1.50, p = 0.13). Note that the two dependent variables in the analyses above were significantly and positively related (Pearson correlation: r = 0.95, p < 0.01), which may, at least partially, explain the similar results of the mixed-model Poisson regressions.

**Figure 3:**
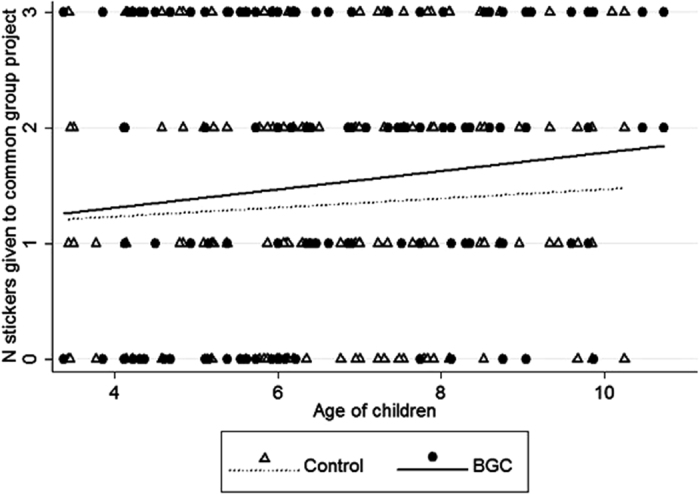
Scatter plot with lines of best fit on the relationship between the age of the children and the number of stickers children gave to the common group project, in experiment #2, in the control (triangles and dotted line) or BGC (circles and continuous line) condition.

In the public goods game (experiment #2) we used a generalised linear mixed model (GLMM) to analyse the number of stickers each child gave to the common group project. The full model, containing experimental condition, age, kinship, familiarity and gender of the child, was significantly different from the null model (likelihood ratio test: chi-square (6) = 13.01, p < 0.05). Supporting our prediction, children gave a greater number of stickers to the common group project in the BGC than in the control condition (Table 3 and Fig. 3; see also Table S2 in the Supplementary Information). Moreover, contribution to the common group project significantly increased with age (Table 3 and Fig. 3). Round of the game, kinship, familiarity and gender of the child did not significantly affect contribution to the common group project (Table 3). Removing from the analyses children who were <six years old, did not change the results of the GLMM: contribution to the common group project was significantly greater in the BGC than in the control condition (coefficient ± SE = 0.30 ± 0.10, z = 2.93, p < 0.01) and by older than younger children (coefficient ± SE = 0.09 ± 0.04, z = 2.54, p < 0.05).

## Discussion

The results of our two experiments support our first hypothesis that there is a positive relationship between BGC and within-group cooperation. A similar result has been found in adult humans^4^,^18^,^24^,^27^ (but see ref. 26) whereas mixed evidence exists for this effect in other species^12^,^13^,^23^. In-group/out-group biases have been found in non-human primates^46^. Therefore, parochial altruism is likely to be a trait shared by various group-living animals but the role of phylogeny, ecology or social structure in explaining its occurrence in different taxa still needs to be clarified.

Our study is the first, to our knowledge, to analyse BGC and within-group cooperation in children as young as three years old up to ten years old. Numeracy and mathematical skills differ significantly between young and old children^43^,^44^. Although all children in our study appeared to understand the rules of the game and did not complain about its complexity, we cannot completely rule out the possibility that children, especially those <six year old, did not fully understand the rules of the experiments and/or the cost/benefit of their decisions, or that children of different age ‘valued’ the stickers differently. Recent work with adults shows that human cooperation in public goods games increases when participants are confused over, or misunderstand how to maximise benefits^47^, although there is no evidence yet that the same factor affects cooperation in children. A cautious interpretation of our findings is required when discussing (see below) the development of parochial altruism and response to out-group threat in children. However, possible age-related differences in the understanding of the rules of the experiments are unlikely to fully explain the positive relationship between BGC and within-group cooperation found in our study, in light of three considerations. First, the use of two experiments, differing in the numeracy skills required to maximise benefits and in the opportunities children had to keep track of their gains and of the other children’s choices, gave similar results (supported by analyses restricted to ≥six years old children), suggesting the experimental design had limited effect on our findings. Second, confusion over resource maximisation in public goods games is expected to affect estimation of overall cooperation (which was not the aim of our study) but should be a less important factor when comparing experimental conditions^48^. Third, and most important, if younger children did not fully understand the rules of the game, and if confusion over the experiment design affects cooperation in children as it does in adults, we would expect greater within-group cooperation from younger than older children (i.e. from children who understood the game the least^47^,^48^); we found the opposite pattern in our data. Note that the age of the children did not significantly differ between the two experimental conditions, showing that children of different age were approximately equally represented in the two conditions.

Our study suggests that the relationship between BGC and within-group cooperation emerges early during the course of human development. Since, in traditional human societies, between-group conflicts usually only involve post-puberty males^1^,^35^, it is unlikely that the evolutionary pressure that led to correlated evolution between BGC and within-group cooperation^17^,^49^ directly acted on the early stages of human development. However, there are significant benefits, and thus strong selection pressure for traits that favour individual discrimination (i.e. care-giver versus unfamiliar subject) and the formation of social bonds with care-giver in children from the first few weeks of their life. For example, the discrimination of faces on the basis on familiarity, phenotype or emotional expression is observed in 5–7 months old children and develops during the neonatal period^50^,^51^,^52^. Such discrimination is of fundamental importance for the neonate to avoid threat/negative stimuli and to attach to care-givers^53^. However, the capacity to differentiate amongst individuals on the basis of the potential costs/benefits they can give, and the tendency to act pro-socially to those who are likely to respond in kind are also fundamental traits for the emergence of in-group/out-group biases and parochial altruism^17^,^41^. We argue that hypothesised selection pressure on human social evolution from an extended period of warfare^20^, which led to correlated evolution between BGC and within-group cooperation, acted on traits that emerge early during development and that may have an older, shared phylogenetic history in altricial mammals. ‘Sensitivity’ to out-group threats might result in innate behavioural (e.g. parochial altruism) and emotional responses (e.g. happiness or shame^25^) that are not necessarily related to decisions and strategies to maximise outcome based on game-theory and the payoff matrix of the experiment^40^,^46^. If so, the increase of within-group cooperation in children in the BGC condition may be a response to the mere presence of an out-group ‘threat’, not related to outcome maximisation. This hypothesis would explain why we obtained an increase of within-group cooperation in experiment #1, where children were kept blind on whether the amount of selfish or cooperative acts was being compared with those of other groups (that is, children did not know whether being selfish or cooperative resulted in wins over other groups). The hypothesis of an innate response to out-group threat is also supported by the observation that social and cultural norms can modify in-group/out-group biases, during development or into adulthood, only to some extent^32^,^54^. For example, extinction procedures do not remove conditioned fear response to faces of people belonging to out-group (i.e. different ethnic group than participant’s own ethnicity), whereas extinction procedures are more effective when removing fear response to faces of people belonging to in-group (i.e. same ethnic group as participant’s own group^55^). In-group/out-group biases in humans appear to be a consequence of in-group favouritism than a response to norm violations^56^.

Unfortunately, a question that our experimental design does not allow us to answer is whether the increase of within-group cooperation in a BGC condition was due to attempts of children to out-perform other groups, to be more cooperative towards their group companions or to relaxation of the incentive to act selfishly^25^. Moreover, our experimental design did not allow us to determine whether the increased within-group cooperation in the BGC condition was due to the description of the competitive scenario or to group labels (i.e. children in the BGC condition were asked to choose a name for their group; see Methods and Supplementary Information) that facilitated group categorisation^36^,^57^.

We found partial support for our second hypothesis: age of the children had a significant and positive relationship with two of our measures of within-group cooperation (i.e. the number of children the player gave stickers to, in experiment #1, and the number of stickers given to the common group project, in experiment #2) but a marginal non-significant relationship with the number of stickers that children gave to their group companions (experiment #1). The effect of age became non-significant when we restricted our analyses to ≥6 years old children but this could be due to the smaller age range considered in those analyses. Apart for the necessary caution to interpret the effect of age in our analyses (see discussion above), the increased within-group cooperation in older children support previous work on the development of altruism, inequity aversion and in-group/out-group biases. Children reach a level of cooperation in public goods game comparable to adults when they are between 6–12 years old^40^. Whilst younger children (i.e. 3–4 years old) often behave selfishly, older children become progressively more concerned about the unequal distribution of resources within their group, more prone to in-group favouritism and parochialism, and more likely to punish free-riders^36^,^37^,^38^,^39^,^41^,^58^. Therefore, within the age range considered in our study, important developmental changes related to pro-sociality and to group membership take place.

In the public goods game (experiment #2), similarly to previous studies^4^,^18^, out-competing other groups resulted in an equal share of the resource gained among group members. Such equal distribution of resources following a successful competitive interaction with another group is unlikely to be frequently observed in real life events. In warfare interactions, for example, whilst the costs may be shared about equally by individuals who take part in the conflict, the benefits of winning a conflict may be skewed in favour of a smaller number of individuals (e.g. leaders/noblemen^1^,^59^). Future experiments on this topic should analyse the role of leadership and unequal distribution of resources following BGC in a public goods game scenario^8^.

Sex of the children and whether they had a familiar or kin child in their group did not affect within-group cooperation. Males take a more active role in warfare than females in various human societies^1^ and thus males should be more responsive to out-group threats^21^. However, our hypothesis that the evolution of traits linked to parochial altruism is rooted into the evolution of infant-care-giver relationship does not predict differences between the sexes. Kinship^7^,^19^ and degree of heterogeneity within a group (measured by, e.g., familiarity, group membership or differences in ethnicity within the group) affect cooperation and response to public goods games^9^,^31^. However, we never had more than two kin/familiar children in each group; the lack of significance for kinship and familiarity may be related to small sample size, especially for experiment #1 (Supplementary Information). Moreover, since in experiment #2 contributions to the common group project were shared equally among children in the group, irrespective to kinship or familiarity, this procedure might have reduced the effect that these two factors play for cooperation.

In conclusion, our study is the first to show that BGC is positively related to within-group cooperation in 3–10 years old children. In-group/out-group biases and parochialism in humans can be explained by our evolutionary past and the potential benefits/costs of BGC. One of the main unexplored areas within this topic remains whether, or to what extent humans maintain in-group/out-group biases despite living in multi-level heterogeneous societies with flexible group membership.

## Methods

### Participants

Participants to this study were 120 typically developing children (60 females and 60 males), between the age of three and ten years old (mean age ± SE: females: 6.48 ± 0.23; males: 6.92 ± 0.24). Data were collected in August 2015 at the Summer Scientists week, an event organised every summer by the School of Psychology, University of Lincoln (see Supplementary Information). This study has been approved by the University of Lincoln Ethics Committee and the data collection has been carried out in accordance to the guidelines of the University of Lincoln; parents/carers of the children who took part in this study signed an informed consent form.

### Experimental design

Each child played the two experiments in a group of four children. Group composition remained the same in the two experiments and no child took part in more than two experiments or was it in more than one group. Each child/group played the two experiments either under a BGC condition or a control condition (no BGC). In the BGC condition, before starting the two experiments, we asked children to choose a name for their group; children in the control condition were not asked to choose a name for their group. Of the 120 children who took part to this study, 14 groups (56 children) played the two experiments under the control condition and 16 groups (64 children) under the BGC condition. Half of the groups, within each condition, played experiment #1 first and the other half played experiment #1 second. The two experiments were separated by approximately 10 minutes and were described to the children as two separate studies. The resource used in the two experiments consisted of 1 × 1 cm stickers depicting a star; at the end of each experiment children could exchange the star stickers they had kept or received from the other children, with a range of reward stickers that they could ‘buy’ according to the rules described in the game instructions (i.e. 3 stickers ‘buy’ one reward sticker; Supplementary Information).

#### Experiment #1

At the start of experiment #1 we read the instructions of the game (Supplementary Information) to the children depending on whether they had been allocated to the BGC or control condition. We told the children that the selection of the player (i.e. the child who was asked to play the game) was random and that the game consisted of an indefinite number of rounds. However, we played four rounds of the game, each child was selected to be the player only once, and we kept the same order of play across groups (i.e. children A played the game first, followed by B, C and D, based on the position of their seat at the experimental table: Supplementary Information).We asked the player to put the stickers they wanted to give to the other children in their group, on the table, in front of the child (children) of their choice and to leave the stickers they wanted to keep, on the table, in front of them. Once the player had made their choice we asked children who had kept or received stickers to put them in the plastic cup that each of them was given and we started another round of the game by selecting another player.

In order to simulate BGC, we told children in the BGC condition that they were going to take part in a competition where their performance during the games was compared to that of other groups. We showed children a fictitious league table on a whiteboard on the wall of the room with the names of the three fictitious groups that were leading the competition at that point. Unlike the BGC condition in experiment #2 (see below), here we did not give children any detail on what criteria we were going to use to compare their performance to that of other groups (e.g. whether, in order to out-compete another group, they had to keep more stickers for themselves or share more stickers with the other children in their group) nor any details on what benefits children would get by out-competing other groups. This procedure allowed younger children to play the game without the need to rely on numeracy and mathematical skills to calculate the consequences of their choices and to maximise their resource gain. Moreover, this design simulated BGC minimising the risk that children implicitly assumed that it was ‘better’ for them to be selfish or cooperative. Finally, our experimental approach in the BGC condition did not rely on a threshold (sensu^26^) that children had to reach in order to out-compete the other groups and gain an additional reward.

#### Experiment #2

Following^18^, we asked children to play a public goods game^4^,^8^,^40^ under a BGC or control condition. At the start of the experiment we read the instructions of the game (Supplementary Information) to the children depending on whether they had been allocated to the BGC or control condition. We told children that the game consisted of an indefinite number of rounds in order to avoid end-game effects; in fact, each group only played three rounds of the experiment. We asked children to put the stickers in the two envelopes (one for the common group project and one for themselves) provided to each of them so that their choice could not be seen by the other children in the group. Once the children had made their choice we opened the envelopes in the common group project box and counted the number of stickers in there. We told children that we were going to multiply the number of stickers in the common group project by a factor α (α = 2) and to divide them equally among the four children. The benefit for a child to give a sticker to the common group project was^18^:


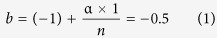


where α is the multiplying factor (α = 2) and n is the number of children in the group (n = 4). Therefore, children were better off keeping all the stickers for themselves than contributing to the common group project, regardless of the choice of the other children in the group to contribute or not.

We asked the children to put all the stickers they got from the common group project and the stickers they kept for themselves in a plastic box, next to them, at the side of the table (the content of the box was not visible to the other children so they could not track how many stickers the other group members had got/kept during the game). This step completed the first round; we started the second round by giving the children three new stickers each and followed the same steps described above until we completed the three rounds of the game.

In order to simulate BGC, we showed children the same fictitious league table with the names of the three fictitious groups as the one used for experiment #1 (see above). Moreover, in experiment #2 we told children that the three groups leading the competition had so far the largest amount of stickers given to the common group project. We told children in the BGC condition that their total number of stickers in the common group project was going to be compared with those of the three leading groups on the whiteboard. We also told children that, if they ended up having a greater number of stickers in the common group project than one of the three leading groups on the whiteboard, they would earn double the difference (β = 2) between the contribution to the common group project of their group and the other group equally shared among them. A contribution of one sticker to the common group project in the BGC did not give children more earning since^18^:


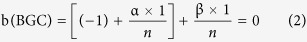


Therefore, ‘winning’ (i.e. having a greater number of stickers in the common group project than one the three leading groups on the whiteboard), ‘drawing’ or ‘losing’ the competition with other groups did not affect the number of stickers children earned in the BGC condition.

### Data analysis

Within the groups, the age of the children was not significantly different in both the BGC (repeated measures ANOVA: F (3, 45) = 0.81, p = 0.49) and in the control conditions (F (3, 39) = 1.01, p = 0.40). The absolute age difference among the four children in each group was 0.56 ± 0.05 years (mean ± SE). Moreover, the average age of the children in each group did not significantly differ between the BGC and control conditions (independent sample t test: t = 0.37, n = 30, p = 0.36), nor was the number of males and females (Mann-Whitney test: z = 0.36, N1 = 56, N2 = 64, p = 0.72). The order of the experiment (whether experiment 1 was run first or second) did not significantly affect within-group cooperation (all p > 0.27) and thus this factor was not included in the analyses presented below.

For experiment #1, our two dependent variables (both ranging from 0 to 3) were the number of stickers that the players gave to children in their group in each round and the number of children who received any stickers from the player. The two dependent variable were analysed in two Poisson regressions where we entered, as independent variables, our two test variables, experimental condition (binomial: BGC or control condition) and age of the children (continuous), together with five control variables: round of the game (1^st^, 2^nd^, 3^rd^ or 4^th^), kinship (binomial: whether the player gave their stickers to kin or not), familiarity (binomial: whether the player gave their stickers to a friend/familiar child or not), gender of the child (binomial: female or male) and group ID.

In experiment #1 we had to discard data on three groups (12 children, 3 groups), allocated to the control condition, because one child in each of these groups left at the start of experiment (after having completed experiment #2; therefore, analyses for experiment #2 are based on the full dataset composed of 120 children). This gave us data on 108 children for experiment #1 (11 groups - 44 children - in the control condition and 16 groups - 64 children-in the BGC condition).

For experiment #2, the number of stickers (ranging from zero to 3 stickers) that each child gave to the common group project in each round was our dependent variable entered in a GLMM with a logarithmic function and Poisson distribution. Experimental condition (binomial: BGC or control condition) and age of the children (continuous) were our test variables in the GLMM, together with five control variables: round of the game (1^st^, 2^nd^ or 3^rd^), kinship (binomial: whether the child had a kin sibling in the group or not), familiarity (binomial: whether the child had a friend/familiar child in the group or not), gender of the child (binomial: female or male). We also entered, as two random factors, the children ID nested into the group ID to control for repeated observations on the same children and for possible differences between groups not related to our independent variables. Following this procedure, the GLMM was based on n = 360 data points (120 children × 3 rounds of the game for each child).

The six independent variables used in the two Poisson regressions and the GLMM were not significantly related to one another (Spearman rank correlation: rho <0.28 for all the correlations and average rho = 0.09). We compared the results of the full models to the null models (i.e. models not containing the independent variables) via likelihood ratio tests. Statistical analyses were performed in Stata v.12.1^60^.

## Additional Information

**How to cite this article:** Majolo, B. and Maréchal, L. Between-group competition elicits within-group cooperation in children. *Sci. Rep.* **7**, 43277; doi: 10.1038/srep43277 (2017).

**Publisher's note:** Springer Nature remains neutral with regard to jurisdictional claims in published maps and institutional affiliations.

## Supplementary Material

Supplementary Information

## Figures and Tables

**Table 1:** Test statistics for experiment #1 for the independent variables entered in the mixed-model Poisson regression on the number of stickers the player gave to children in their group.

Variables	Coefficient ± SE	95% CIs	z	P
Condition	0.63 ± 0.30	0.04–1.23	2.08	<0.05
Age	0.12 ± 0.06	0.01–0.23	2.04	<0.05
Gender	−0.16 ± 0.23	−0.60–0.29	−0. 70	0.49
Intercept	−1.09 ± 0.48	−2.04–−0.14	−2.26	<0.05

Unlike the other two models (Tables 2 and 3), in analysis we excluded three control variables (round of the game, kinship and familiarity) in order to reach convergence.

**Table 2:** Test statistics for experiment #1 for the independent variables entered in the mixed-model Poisson regression on the number of children the player gave stickers to in their group.

Variables	Coefficient ± SE	95% CIs	z	P
Condition	0.87 ± 0.34	0.21–1.53	2.59	<0.01
Age	0.18 ± 0.06	0.05–0.30	2.74	<0.01
Round	0.04 ± 0.09	−0.14–0.21	0.41	0.68
Kinship	−0.14 ± 0.29	−0.71–0.42	−0.49	0.62
Familiarity	0.02 ± 0.31	−0.58–0.62	0.06	0.95
Gender	−0.18 ± 0.24	−0.64–0.29	−0.74	0.46
Intercept	−1.78 ± 0.62	−3.00–−0.56	−2.86	<0.01

**Table 3:** Test statistics for experiment #2 for the independent variables entered in the GLMM on children’s contribution to the common group project.

Variables	Coefficient ± SE	95% CIs	z	P
Condition	0.25 ± 0.09	0.07–0.43	2.67	<0.01
Age	0.05 ± 0.03	0.00–0.10	2.08	<0.05
Round	0.03 ± 0.05	−0.07–0.15	0.63	0.53
Kinship	0.05 ± 0.09	−0.14–0.23	0.49	0.63
Familiarity	−0.03 ± 0.09	−0.21–0.14	−0.39	0.70
Gender	0.01 ± 0.09	−0.15–0.18	0. 16	0.88
Intercept	−0.14 ± 0.22	−0.57–0.29	−0.64	0.52
