# Supplementary material for: Between-group competition elicits within-group cooperation in children
Source: Sci Rep. 2017 Feb 24;7:43277. doi: 10.1038/srep43277 (PMC5324140; doi:10.1038/srep43277)
Supplement: Supplementary Information [file srep43277-s1.pdf]

Between-group competition elicits within-group cooperation in children

Bonaventura Majolo and Laëtitia Maréchal

School of Psychology, University of Lincoln, Lincoln LN6 7TS, United Kingdom

Supplementary Methods

We collected data at the Summer Scientists week, at the School of Psychology, University of Lincoln. Children between the age of three and ten years old can attend the Summer Scientists week and take part in various research projects and recreational activities. The five days of the Summer Scientists week are divided in two sessions each (i.e. morning and afternoon session); each child participating to this event can only attend one session.

We used two experiments to analyse if within-group cooperation is related to between-group competition. Experiment #1 was a simple experiment where children could decide whether they wanted to give some/all of their stickers to one or more children of their choice in the group. Experiment #2 was a public goods game, previously used with adult humans (Puurtinen and Mappes (2009), and adapted it for children. In comparison to the study run by Puurtinen and Mappes (2009), experiment #2 differed in four main aspects. First, similarly to experiment #1, we used a between-subject design (i.e. children played the BGC or control condition) and let children to only play three rounds of a public goods game (instead of a within-subject design with ten rounds in each condition; Puurtinen and Mappes 2009). Second, children played a public goods game in a group with face-to-face interactions between group members rather than interacting via computer. Third, similarly to other studies (Prencipe & Zelazo, 2005; Moore 2009) we used stickers as the resource at stake during the public goods game, instead of monetary units (Puurtinen and Mappes 2009), because children have a vague understanding of the value of money up until they reach 8-10 years of age (Berti et al. 1981). We did not use candies (e.g. Fehr et al. 2008) to more easily get parent approval. Previous research that has compared the use of stickers and candies in similar procedures has found no difference in performance for the two reward types (Prencipe & Zelazo, 2005). Fourth, we gave children three stickers per round instead of a larger amount of resource (e.g. 20 monetary units; Puurtinen and Mappes 2009) because younger children might struggle to keep track of the distribution of large quantities of the resource at stake.

At the beginning of each half-day session, the Summer Scientist support team asked parents/carers to sign the consent form for their children to take part in our study. They also asked parents/carers to tell us the date of birth and gender of their child/children, and whether the child had any special needs; each child was given a unique ID code that they could use to take part in the various project and activities run during the session. We allocated children to different time slots and informed their parents about the time of the experiment for their children and the location of the experimental room. When forming each group of four children, we minimised differences within the group (i.e. depending on the number of children for each gender and age registered for the half-day session), and between groups allocated to the control or BGC condition, for the following variables (in decreasing order of importance): 1) age of the children (i.e. same age of children within each group; same number of children of each age group in the control and BGC condition; Table S1); 2) presence of kin (i.e. groups with no kin preferred over groups with kin; same number of children with kin in their group in the control and BGC condition); 3) familiarity (i.e. groups with no familiar children preferred over groups with familiar children; same number of children with familiar children in their group in the control and BGC condition); 4) gender (i.e. same number of males/females within each group; same number of males/females in the control and BGC condition). We never had more than two kin/familiar children in each group. We had 13 groups with familiar children (7 groups, 50%, in the control, and 6 groups, 37.5%, in the BGC condition) and 14 groups with kin (9 groups, 64.3%, in the control, and 5 groups, 31.3%, in the BGC condition). In experiment #1, the number of rounds in which the player had a familiar child or kin in the group, and also decided to give stickers to other children, was small (kinship: 14.3%, familiarity: 20.4%) so we could not perform additional

analyses to test the effect of kinship and familiarity on the two dependent variables in experiment #1.

Within each experimental condition, half of the groups played experiment #1 first and the other half played experiment #2 second. Children were given around 10 minutes to rest between the two experiments.

At the beginning of each experimental session we greeted and thanked the parents and their children for their participation. We asked the children to seat at the four sides of a table at the centre of the experimental room (parents waited outside). One of us (LM) led the experiment and collected data with the help of two assistants. Once the children at the experimental table, we recorded the ID code of each child (which was used after the experiment to get data on their date of birth, gender and whether they had special needs) and their position at the table. We described the game instructions to the children following the text below, depending on whether we had allocated the group of four children to the control or between-group competition condition, and on whether they were going to play experiment #1 or experiment #2 first. At the end of each experiment we let children to exchange their stickers with a range of reward stickers that they could 'buy' according to the rules described in the game instructions (3 star stickers 'buy' one reward sticker). A large range of reward stickers, suitable for children of different age and gender, was available for the children to choose on. If children had to play the second experiment (experiment #1 or #2 depending on which experiment they had played first), we gave them approximately 10 minutes to rest before starting the second experiment play. Once the break was over, we asked children to seat at the same position at the table where they were sitting for the first experiment and we read them the game instructions. At the end of the second experiment children could exchange the star stickers then had kept/gained in the second experiment with additional reward stickers, following the rules described above. Once the two experiments had been completed, we debriefed the children and their parents about the aims of the study and thanked them for their participation. Descriptive statistics on within-group cooperation performed by children in each round of the two experiments is shown in Table S2 below.

In order to control whether our dependent variables were over-dispersed, we compared the mean to the variance for each dependent variable and then we visually inspected the frequency distribution of the data. For experiment #1, the variance was less than two times the mean (number of stickers the player gave to children in their group: mean=1.09, variance=1.79; number of children the player gave stickers to in their group: mean=0.99, variance=1.60), indicating little over-dispersion. However, visual inspection of the frequency distribution of these two variables showed a marginal skew in favour of zero values. Thus, to further control for the effect of over-dispersion on our results for experiment #1, we ran two mixed-model negative binomial regressions (MMNBRs) with the same fixed and factors used in the two mixed-model Poisson regressions. The two MMNBRs gave very similar results to those obtained with the mixed-model Poisson regression. The number of stickers given, and the number of recipients of stickers, were significantly greater in the BGC than in the control condition (p=0.026 ad p=0.008, respectively). The relationship between age of the children and the number of stickers given was marginally non-significant (p=0.071) whereas older children gave stickers to a larger number of recipients than younger children (p=0.018). The other fixed factors had no significant effect. Moreover, the likelihood-ratio tests of the over-dispersion parameter alpha linked to each the two MMNBRs were not significant (both p>0.49). Therefore, we concluded that over-dispersion did not affect the results of experiment #1. For experiment #2, the variance was lower than the mean of stickers given by children (mean=1.53, variance=1.28) and visual inspection of the frequency distribution of this variable showed no skew. Thus, we did not run any additional check on over-dispersion for experiment #2

Table S1: number of children and their average age in the control and the between-group competition (BGC) conditions.

Age category (years old)	Control		BGC	
	Mean age ± SE	N children	Mean age ± SE	N children
3	3.40 ± 0.01	2	3.38	1
4	4.35 ± 0.01	4	4.01 ± 0.04	7
5	4.99 ± 0.07	7	5.01 ± 0.03	9
6	6.06 ± 0.05	13	5.98 ± 0.03	17
7	7.07 ± 0.07	10	6.99 ± 0.03	9
8	7.92 ± 0.03	10	7.95 ± 0.06	10
9	8.80 ± 0.04	6	8.91 ± 0.10	6
10	10.28 ± 0.08	4	9.73 ± 0.03	5

Table S2: Mean (± SE) number of stickers given (experiment #1 and #2), and of recipients of the stickers (experiment #2), per round, in the control and the between-group competition (BGC) conditions.

Experiment #1: number of stickers that the player gave to children in their group		Control	BGC
Round	1	0.46 ± 0.31	1.38 ± 0.35
	2	0.82 ± 0.38	1.13 ± 0.35
	3	0.55 ± 0.31	1.63 ± 0.38
	4	1.00 ± 0.38	1.31 ± 0.35
Experiment #1: number of children that the player gave stickers to in their group			
Round	1	0.46 ± 0.31	1.38 ± 0.35
	2	0.55 ± 0.28	1.00 ± 0.33
	3	0.45 ± 0.25	1.56 ± 0.37
	4	0.64 ± 0.28	1.31 ± 0.35
Experiment #2: number of stickers that children gave to the common group project			
Round	1	1.43 ± 0.25	1.56 ± 0.24
	2	1.14 ± 0.29	1.50 ± 0.30
	3	1.43 ± 0.34	1.81 ± 0.21

References

Berti, A.E., & Bombi, A.S. (1981). The development of the concept of money and its value: A longitudinal study. *Child Development*, 1179-1182.

Moore, C. (2009). Fairness in children's resource allocation depends on the recipient. *Psychological Science*, 20(8), 944-948.

Prencipe, A., & Zelazo, P.D. (2005). Development of affective decision making for self and other: Evidence for the integration of first and third-person perspectives. *Psychological Science,* 16, 501–505.

Puurtinen, M., & Mappes, T. (2009). Between-group competition and human cooperation. *Proceedings of the Royal Society of London B: Biological Sciences*, 276(1655), 355-360.

Experiment 1 - Game instructions for the control condition

1. You are going to play a game. You will be in this same group during the whole game. Please pay careful attention to the game instructions. If you have any question, please raise your hand. Otherwise, please be quiet and listen carefully, just like you would to your teacher in school.
2. We will randomly select one of you as the player of this game. If you are the selected child (the player), we will give you three star stickers. You have to decide whether you are going to keep the star stickers for yourself and/or you are going to give some/all of stickers to one or more children in the group. If you want to keep some/all stickers for yourself, you can leave them next to you on the table. If you want to give some/all of stickers to one or more children in the group, you can pass the stickers to the other child/children of your choice and leave the stickers in front of them on the table. Please do whatever you wish, either share your stickers or keep them; both are good options.
3. Once you have made your choice, we will ask children who have stickers to put them in the plastic glass provided so to avoid confusion with the stickers used in the next round. After this, we will start another round of the game and randomly select another player.
4. You can keep the stickers, that you have kept for yourself and/or that you have received from other children, next to you on the table. We will play an indefinite number of rounds of this game and you might be ‘the player’ once, more than once or never.
5. Once this game is completed, you can count all the stickers you kept or received from other children and exchange your star stickers with the reward stickers of your choice: you need three start stickers to get one reward sticker.
6. If you have any question, please raise your hand now. Otherwise, let’s start the game.

Experiment 1 - Game instructions for the between-group competition condition (text underlined is for instructions only used in the between-group competition condition)

1. You are going to play a game. You will be in this same group during the whole game. Please pay careful attention to the game instructions. If you have any question, please raise your hand. Otherwise, please be quiet and listen carefully, just like you would to your teacher in school.
2. We will randomly select one of you as the player of this game. If you are the selected child (the player), we will give you three star stickers. You have to decide whether you are going to keep the star stickers for yourself and/or you are going to give some/all of stickers to one or more children in the group. If you want to keep some/all stickers for yourself, you can leave them next to you on the table. If you want to give some/all of stickers to one or more children in the group, you can pass the stickers to the other child/children of your choice and leave the stickers in front of them on the table. Please do whatever you wish, either share your stickers or keep them; both are good options.
3. Once you have made your choice, we will ask children who have stickers to put them in the plastic glass provided so to avoid confusion with the stickers used in the next round.
4. You can keep the stickers, that you have kept for yourself and/or that you have received from other children, next to you on the table. We will play an indefinite number of rounds of this game and you might be ‘the player’ once, more than once or never.
5. <u>We are running a tournament where we compare what you do during this game with what children in other groups have done. The top three groups at the moment in our tournament are White, Black and Grey, as you can see on the league table on the wall. Let’s see if you can beat these three top groups.</u>
6. Once this game is completed, you can count all the stickers you kept or received from other children and exchange your star stickers with the reward stickers of your choice: you need three start stickers to get one reward sticker.
7. If you have any question, please raise your hand now. Otherwise, let’s start the game.

Experiment 2 - Game instructions for the control condition

1. You are going to play a game consisting of various rounds. You will be in this same group during the whole game. Please pay careful attention to the game instructions, because the clearer the game instructions are the more star stickers you can earn, and the more star stickers you can earn the more reward stickers you can get at the end of the game). If you have any question, please raise your hand. Otherwise, please be quiet and listen carefully, just like you would to your teacher in school.
2. We will give each of you three star stickers before playing each round of the game. You have to decide whether you are going to keep the star stickers for yourself and/or you are going to give some/all of stickers to the common group project.
3. Next to you, you have two envelopes. The envelop that has “you” written on is where you can put the stickers you want to keep for yourself (if any) whereas the envelope that has “group” written on is where you put the stickers you want to give to the common group project.
4. Your choices have to be kept secret so please do not show whether and how many stickers you put in each envelope, and put the group envelope in the common group bowl even if it has no stickers in it. The best way to keep your decision secret is for you to move away from the table, at your side of the room, put the stickers in the two envelopes according to your decision, and then return to the table to continue the game. We do not think it would be better for you to give the stickers to the common group project or better to keep them for yourself.
5. When I clap my hands, you will need to make your choice. Once you have put the stickers in the two envelopes, you can leave the “you” envelop next to you and the “group” envelope in the common group project box at the centre of the table. Please put the “group” envelope in the box even if it doesn’t contain any stickers and remember your choice is secret.
6. Once all of you have made your choice and positioned the two envelopes, we will open the “group” envelopes in the common group project box and count them. We will multiply the stickers in the common group project box by two and divide them equally among you, no matter how many (if any) stickers you have given to the common group project. This means that if, for example, in the common group project box there are 4 stickers in total, we will multiply this number by two, giving us eight stickers in total; each of you will receive two stickers from the common group project no matter how many stickers you put in the common group project box.
7. Once you receive the stickers from the common group project you can put them in the container at the side of your chair, together with the stickers you kept for yourself (the ones you put in the “you” envelope). Please remember that it is a secret how many stickers you have so do not show the content of this box to the other children.
8. Once these steps have been completed, we will give each of you three new star stickers and we will play another round following the same steps of the first round. We will play an indefinite number of rounds before completing the game.
9. At the end of each game, you can count all the stickers you have in the box at the side of your chair and exchange your star stickers with the reward stickers of your choice: you need three start stickers to get one reward sticker.
10. If you have any question, please raise your hand now. Otherwise, let’s start the game.

Experiment 2 - Game instructions for the between-group competition condition (text underlined is for instructions only used in the between-group competition condition)

1. You are going to play a game consisting of various rounds. You will be in this same group during the whole game. Please pay careful attention to the game instructions, because the clearer the game instructions are the more star stickers you can earn, and the more star stickers you can earn the more reward stickers you can get at the end of the game). If you have any question, please raise your hand. Otherwise, please be quiet and listen carefully, just like you would to your teacher in school.
2. We will give each of you three star stickers before playing each round of the game. You have to decide whether you are going to keep the star stickers for yourself and/or you are going to give some/all of stickers to the common group project.
3. Next to you, you have two envelopes. The envelop that has "you" written on is where you can put the stickers you want to keep for yourself (if any) whereas the envelope that has "group" written on is where you put the stickers you want to give to the common group project.
4. Your choices have to be kept secret so please do not show whether and how many stickers you put in each envelope, and put the group envelope in the common group bowl even if it has no stickers in it. The best way to keep your decision secret is for you to move away from the table, at your side of the room, put the stickers in the two envelopes according to your decision, and then return to the table to continue the game. We do not think it would be better for you to give the stickers to the common group project or better to keep them for yourself.
5. When I clap my hands, you will need to make your choice. Once you have put the stickers in the two envelopes, you can leave the "you" envelop next to you and the "group" envelope in the common group project box at the centre of the table. Please put the "group" envelope in the box even if it doesn't contain any stickers and remember your choice is secret.
6. Once all of you have made your choice and positioned the two envelopes, we will open the "group" envelopes in the common group project box and count them. We will multiply the stickers in the common group project box by two and divide them equally among you, no matter how many (if any) stickers you have given to the common group project. This means that if, for example, in the common group project box there are 4 stickers in total, we will multiply this number by two, giving us eight stickers in total; each of you will receive two stickers from the common group project no matter how many stickers you put in the common group project box.
7. <u>We are running a tournament where we calculate children's contribution to the common group project. The top three groups at the moment in our tournament are White, Black and Grey, as you can see on the league table on the wall. You have been selected to take part in this tournament. At the end of the game, if your group ends up being one of the top three groups, we will calculate the difference between the total contribution to the common group project of your group and that of the group standing immediately below yours. We will double that difference and distribute the stickers equally among you. For example, if you end up being above the White group and the difference between your group and the White group is 4 stickers in total, we will multiply this number by two, giving us eight stickers in total; each of you will receive two stickers from the common group project no matter how many stickers you put in the common group project box.</u>
8. Once you receive the stickers from the common group project you can put them in the container at the side of your chair, together with the stickers you kept for yourself (the ones you put in the "you" envelope). Please remember that it is a secret how many stickers you have so do not show the content of this box to the other children.

9. Once these steps have been completed, we will give each of you three new star stickers and we will play another round following the same steps of the first round. We will play an indefinite number of rounds before completing the game.
10. At the end of each game, you can count all the stickers you have in the box at the side of your chair and exchange your star stickers with the reward stickers of your choice: you need three start stickers to get one reward sticker.
11. If you have any question, please raise your hand now. Otherwise, let’s start the game.
